# Infection with Classical Swine Fever Virus Induces Expression of Type III Interferons and Activates Innate Immune Signaling

**DOI:** 10.3389/fmicb.2017.02558

**Published:** 2017-12-19

**Authors:** Binxiang Cai, Qingling Bai, Xiaojuan Chi, Mohsan U. Goraya, Long Wang, Song Wang, Biao Chen, Ji-Long Chen

**Affiliations:** ^1^Key Laboratory of Fujian-Taiwan Animal Pathogen Biology, College of Animal Sciences, Fujian Agriculture and Forestry University, Fuzhou, China; ^2^CAS Key Laboratory of Pathogenic Microbiology and Immunology, Institute of Microbiology, Chinese Academy of Sciences, Beijing, China

**Keywords:** innate immunity, type III interferons, classical swine fever virus, interferon stimulated genes, STAT1

## Abstract

Classical swine fever virus (CSFV) commonly infects the lymphatic tissues and immune cells of pigs and could cause a lethal disease in the animals. The process and release of cytokines like type III interferons (IFNs) is one of the important responses of the host innate immunity to viral infection. However, little information is available about type III IFN response to the CSFV infection. In this study, we investigated the expression of type III IFNs including interleukin-28B (IL-28B) and IL-29 in PK-15 cells and pigs following CSFV infection. We found that infection with CSFV was able to induce expression of IL-28B and IL-29 in PK-15 cells, although the increased levels of type III IFNs were limited. Importantly, up-regulation of IL-28B and IL-29 was further observed in CSFV infected animal tissues. The production of IL-28B and IL-29 was reduced by the inactivation of NF-κB in cells, indicating that activated NF-κB is required for efficient expression of type III IFNs induced by CSFV. Moreover, our experiments demonstrated that infection with CSFV strongly stimulated the downstream of STAT1 signaling *in vitro* and *in vivo*. In addition, several critical IFN-stimulated genes (ISGs) including IFITM3, OASL, OAS1, and ISG15 were significantly upregulated at both mRNA and protein levels in PK-15 cells and infected pigs. Together, these results reveal that CSFV can trigger host antiviral immune responses including production of type III IFNs, activation of STAT1, and induction of some critical ISGs.

## Introduction

Host innate immunity against viruses is stimulated by sensing the viral infection via pattern recognition receptors (PRRs). Such receptors include Toll-like receptors (TLRs), NOD like receptors and the cytosolic retinoic acid-inducible gene I (RIG-I) and melanoma differentiation-associated protein 5 (MDA-5), which recognize different conserved microbial structures and molecules, such as single-stranded and double-stranded viral RNA, collectively termed as pathogen associated molecular patterns (PAMPs). The interaction between host PRRs and viral PAMPs is a critical step for initiation of innate immune response. Upon detecting PAMPs, PRRs activate the intracellular signaling pathways that regulate both immediate innate and subsequent adaptive immunity (Iwasaki and Medzhitov, 2010; Goraya et al., 2015). A vital role of innate antiviral response is the rapid induction of interferons (IFNs), which further stimulates a cascade of IFN-stimulated genes (ISGs) whose expression shapes antiviral and immune-modulatory actions that help to limit the viral infection and replication (Takaoka and Yanai, 2006). It is known that expression of type I and type III IFNs depends upon the activation of interferon regulatory factor-3 (IRF-3), IRF-7 and nuclear factor-κB (NF-κB) via TLRs and RIG-I dependent signaling pathways (Janeway and Medzhitov, 2002). These transcription factors are also involved in regulating production of other cytokines and chemokines. Type III IFNs consist of three members in human, named as interleukin-29 (IL-29), IL-28A, and IL-28B, also known as IFN-λ1, IFN-λ2, and IFN-λ3, respectively. Type I and type III IFNs are considered to be the most important antiviral molecules of innate immune response against viral infection (Borden et al., 2007; Wang et al., 2014; Wei et al., 2014). However, little is known about how type III IFNs respond to classical swine fever virus (CSFV) infection.

Classical swine fever virus (previously called hog cholera virus) belongs to the genus *Pestivirus* of *Flaviviridae* family. CSFV is a spherical viral particle, about 40–60 nm in diameter, has positive single-strand RNA (ssRNA) genome of 12.3 kb surrounded by lipid bilayer (Meyers et al., 1989). The ssRNA of the virus carries a large open reading frame (ORF) and encodes a single polyprotein that is co- and post-translationally cleaved to produce 12 proteins by cellular and viral proteases: N-terminal auto-protease (N^pro^), C, E^rns^, E1, E2, p7, NS2, NS3, NS4A, NS4B, NS5A, and NS5B (Meyers and Thiel, 1996). CSFV is a highly infectious virus responsible for a contagious infection known as classical swine fever (CSF) in pigs. Clinical symptoms and severity of CSF vary according to different strains of the virus, age of the host and the immune status of the herd (Terpstra, 1987; Moennig et al., 2003). After establishing an infection in the tonsils, CSFV invades into host lymphatic organs through blood, with lethal outcome including death of piglets within the infected populations (Susa et al., 1992; Rossi et al., 2011). CSFV is commonly found in T cells, B cells and monocytes (Qu et al., 2001). Persistence of CSFV in lymphoid tissues is considered to be the most significant feature of CSFV infection, which leads to the acquired immunosuppression condition and is associated with pathogenesis of the virus (Summerfield et al., 1998; Ambagala et al., 2005). In addition, it is thought that virulence of CSFV is associated with mutations in several viral proteins including N^pro^, C, E^rns^, E1, E2, p7, and NS4B, which are related to viral invasion, uncoating, replication and interaction with cellular factors (Leifer et al., 2013; Ji et al., 2015).

Two crucial PAMPs can be sensed by cellular receptors during CSFV infection: one is the viral genomic ssRNA and the second is double-strand RNA (dsRNA), an intermediate replication product of the virus. The host TLR-3, 7, and 8 are responsible for sensing the RNA viruses (Kawai and Akira, 2006). TLR-7 binds with the ssRNA derived from CSFV and TLR-3 senses the replication intermediate dsRNAs (Diebold et al., 2004; Sen and Sarkar, 2005; Chen et al., 2015). Intracellular detection of ssRNA by TLR-7 triggers the activation of transcription factors IRF-7 and NF-κB, which ultimately initiate the expression of type I IFNs including IFNα and IFNβ (Lund et al., 2004; Cao et al., 2015). TLR-3 signaling activates the transcription factor IRF-3, which induces expression of the IFN-β and type III IFNs upon its entrance into the nucleus of cells (Kawai and Akira, 2006; Chen et al., 2015). It has been established that the presence of viral RNA in the cytoplasm can be detected by cellular RIG-I and MDA-5 (Yoneyama et al., 2005). The interaction between viral dsRNA and RIG-I or MDA-5 causes robust expression of IRF-3 dependent IFNs (Chen et al., 2015). In turn, IFNs can stimulate the gene expression of IRF-7, which results in an enhanced expression of type I IFNs and induces the transcription of antiviral proteins, such as ISG15, interferon-induced transmembrane proteins (IFITMs) and Mx A (Ouyang et al., 2014).

Although progress has been made in understanding host immune response to CSFV infection and immunosuppression caused by the virus, activation of innate immune signaling during CSFV infection and mechanisms underlying the innate immunity against CSFV are not fully understood. In particular, it is still unclear whether type III IFNs is induced by CSFV infection, and little is known about activation of STAT1 and how some critical ISGs are regulated during CSFV infection *in vitro* and *in vivo*. In this study, we analyzed the expression of type III IFNs and some critical ISGs in PK-15 cells infected with CSFV. Furthermore, these important molecules involved in host innate immunity were examined in pigs after CSFV infection. We observed that mRNA levels for IL-28B and Il-29 were increased both *in vitro* and *in vivo* after the infection with CSFV. Furthermore, CSFV infection greatly activated STAT1 and increased the expression of different ISGs, including IFITM3, ISG15, OAS1, and OASL. Interestingly, inactivation of NF-κB significantly reduced the expression of IL-28B and IL-29. These results demonstrate that CSFV can induce host innate antiviral response involving the expression of Type III IFNs and some critical ISGs.

## Materials and Methods

### Antibodies and Reagents

The primary antibodies used in this study included anti-phospho-STAT-1 (Cell Signaling Technology, #7649), anti-STAT-1 (Santa Cruz Biotechnology, sc-346), anti-IκBα (Beijing Golden Bridge Biotechnology, ZS-371), anti-ISG15 (Protein Biotechnology, cat No.: 15981-1-AP), anti-IFITM3 (Protein Biotechnology, cat No.: 11714-1-AP), and anti-β-actin (Transgen Biotechnology, HC201). The pharmacological NF-κB inhibitor BAY 11-7082 was purchased from Merck (Darmstadt, Germany).

### Virus and Cells

Classical swine fever virus Shimen strain was obtained from Prof. Jinding Chen (The South China Agriculture University, Guangzhou, China). To determine the virus titers, swine kidney PK-15 cells were cultured in 96 well plates and inoculated with 10 fold serial dilutions of CSFV Shimen strain. After infection, cells were incubated at 37°C for 5 days. Then, PK-15 cells were fixed with 80% acetone at -20°C for 30 min, and viruses were detected by an immunofluorescence assay using mouse anti-CSFV E2 antibody and FITC-conjugated goat anti-mouse secondary antibody. Virus titers were calculated according to Kaerber Knoetig (Knoetig et al., 1999) and expressed as 50% tissue culture infectious doses (TCID_50_) per milliliter. The multiplicity of infection (MOI) was calculated based on TCID_50_. 293T cells and swine kidney cell line PK-15 were purchased from American Type Culture Collection (Manassas, VA, United States). 293T cells and PK-15 cells were maintained in complete Dulbecco’s modified Eagle’s medium (DMEM) supplemented with 10% fetal bovine serum (FBS) and 1% antibiotics. All cells were incubated at 37°C with 5% CO_2_.

### Viral Infection

PK-15 cells were grown to approximately 80% confluence in cell culture plates and then infected with CSFV at an MOI of 1∼3. The mock cells were treated with the blood of healthy pigs. Then cells were placed at 37°C with 5% CO_2_ for 1 h. Later, cells were washed three times with PBS and cultured in DMEM supplemented with 2% FBS at 37°C with 5% CO_2_. Cultured cells were harvested at different time points. Total RNA and proteins were isolated from the cells and further used for qRT-PCR, PCR and Western blotting. For *in vivo* experiments, five pigs (40 days old) were purchased from a farm, which was CSFV-negative. All pigs were infected by intramuscularly injecting 0.1 ml (TCID50 = 10^-4.25^/0.1 ml) of CSFV Shimen strain or mock per pig. All pigs were reared under the same conditions. At 5 days post-infection, lymphoid tissues (spleen, tonsils, inguinal lymph node, sub-maxillary lymph node, and mesenteric lymph node) of infected or control animals were collected for further studies.

### RT-PCR and Quantitative Real-time PCR

Total RNA was isolated from cultured cells and lymphoid tissues of pigs using TRIzol reagent (Invitrogen, Carlsbad, CA, United States) and reverse transcribed into cDNA utilizing M-MLV Reverse Transcriptase (Promega, United States) according to the manufacturer’s instructions. The cDNA was analyzed by qRT-PCR using TransStart Green qPCR SuperMix (TransGen Biotech) and PCR using rTaq DNA polymerase (Takara Bio). The amplified products by PCR were resolved on 1% agarose gels, and when necessary, the intensity of bands was analyzed using Quantity One software (Bio-Rad, United States), as previously described (Chen et al., 2015). The primers specific for CSFV E2, pig IL-28B, IL-29, OAS1, OASL, IFITM3, ISG15, and β-actin (the reference housekeeping gene for internal standardization) were designed using the Primer 5 software (**Table 1**). The data of qRT-PCR analysis were shown in normalized ratios which was auto-calculated using ΔΔC_T_ method by LightCycler system (Roche, Switzerland).

**Table 1:** Primer sequences used in this study.

Primer name	Primer sequence (5′–3′)
β-actin-F	GACCTGACCGACTACCTCAT
β-actin-R	CGTAGAGGTCCTTCCTGATGT
IL28B-F	ACGCCTGACAAGACCGAAG
IL28B-R	GCAGTTCCAGTCCTCCAAGA
IL29-F	CATGGGCCAGTTCCAATCTCT
IL29-R	CTGATGCAAGCCTGAAGTTCG
OASL-F	CCCACAAGGAGTGTAAAGAAGA
OASL-R	GGCCTCAATCAGATCCACATAG
OAS1-F	GGAAGCCATCGACATCGTCT
OAS1-R	GGGCAGGACATCAAACTCCA
IFITM3-F	CATATGAGATGCTCAAGGAGGAG
IFITM3-R	CAGTGGTGCAAACGATGATG
ISG15-F	GTTGATGGTGCAAAGCTTCAG
ISG15-R	CACATAGGCTTGAGGTCATACTC
Mx1-F	CCCAGATCTGACCCTCATAGA
Mx1-R	CCTCCAGAAGATCCCTGAAATG
CSFV-E2-F	CGGCAACACAACTGTCAAGG
CSFV-E2-R	AGCGGCGAGTTGTTCTGTTA

### Enzyme-Linked Immunosorbent Assay (ELISA)

Cell culture supernatants were collected from the culture medium of PK-15 cells infected with CSFV Shimen strain, as a source of virus-induced cytokines. To quantify production of IL-28B and IL-29 by host cells, cell culture supernatants from virus infected cells was harvested and examined by ELISA using the ready SET-Go of pig IL-28B or IL-29 analysis kit (eBioscience, San Diego, CA, United States) according to manufacturer’s instruction.

### Western Blotting

Western blotting was performed as described previously (Li et al., 2015). Briefly, PK-15 cells were incubated on ice with RIPA lysis buffer (Cell Signaling Technology, United States, #9803) containing 1 mM PMSF (Beyotim, ST506) for 30 min. The lysates were clarified by centrifugation at 12,000 rpm for 10 min at 4°C, and the protein concentration was quantified by the BCA protein assay kit (Beyotim, P0012). Equal amounts of protein samples were separated on 12% SDS-PAGE gels, transferred onto NC membranes (Merck Millipore, HATF00010) and blocked with 5% (w/v) milkpowder in Tris-buffered saline (pH 7.4, TBS) for 2 h at room temperature. The membranes were then probed with indicated primary antibodies for 2.5 h at room temperature. The primary antibodies used in Western blotting included anti-phospho-STAT-1 (dilution 1:2000), anti-STAT-1 (dilution 1:5000), anti-IκBα (dilution 1:1000), anti-ISG15 (dilution 1:1000), anti-IFITM3 (dilution 1:1000), and anti-β-actin (dilution 1:2000). Next, the membranes were washed three times in TBS, followed by incubation with appropriate secondary antibodies conjugated to HRP at room temperature for 2 h. The signals were detected by the ProteinSimple FluorChem M system (ProteinSimple, United States) after incubation with ECL Plus (Thermo Fisher Scientific, 34095).

### Statistical Analysis

Results were shown as mean values ± standard error (mean ± SE). Statistical significance was determined by Student’s *t*-test analysis. A level of *P* < 0.05 was considered to be significant.

### Ethics Statement

The animal protocols used in this study were approved by the Research Ethics Committee of College of Animal Science, Fujian Agriculture and Forestry University (Permit Number PZCASFAFU2014003). The procedures were carried out in accordance with the approved guidelines and the regulations for the Administration of Affairs Concerning Experimental Animals approved by the State Council of China.

## Results

### Expression of Type III IFNs Is Upregulated in PK-15 Cells during CSFV Infection

**FIGURE 1:**
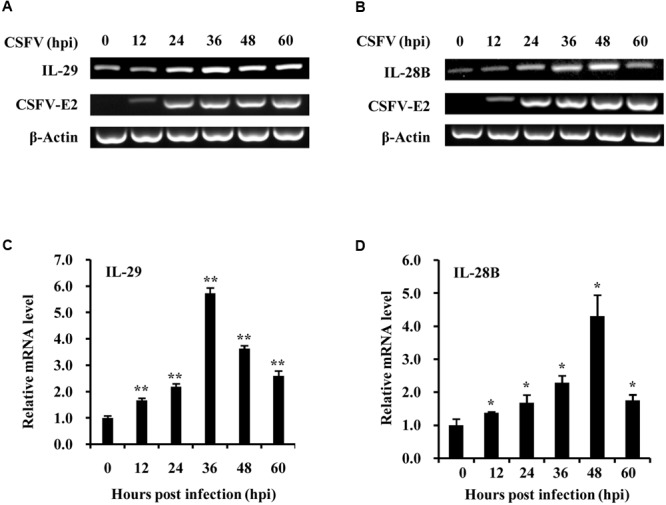
Classical swine fever virus (CSFV) infection induces the expression of type III IFNs in PK-15 cells. **(A,B)** PK-15 cells were infected with or without CSFV Shimen strain and harvested at the indicated time. RT-PCR analysis was performed to determine the expression of CSFV structural protein gene E2, IL-29 **(A)** and IL-28B **(B)**. The CSFV E2 was used to indicate the virus infection and replication. Quantitative real-time PCR was further performed to examine the mRNA levels of Type III IFNs, IL-29 **(C)** and IL-28B **(D)** in PK-15 cells infected with CSFV for indicated time. Plotted are the average results from three independent experiments. ^∗^*P* < 0.05, ^∗∗^*P* < 0.01.

To better understand host innate immune response to CSFV infection, we investigated the cellular transcriptional response of type III IFNs, important components of innate immunity, during CSFV infection of PK-15 swine kidney cells. For this, PK-15 cells were infected with CSFV Shimen strain, and the samples were harvested at different time points of post-infection and examined for expression of CSFV gene and type III IFNs. CSFV was well-replicated in the cells as indicated by robust expression of CSFV E2 gene that encodes one of CSFV structural protein (**Figures 1A,B** and Supplementary Figure S1). Importantly, we observed that expression of swine IL-28B and IL-29, two members of type III IFN family in pig, was clearly upregulated during CSFV infection (**Figures 1A,B**). The highest mRNA levels of IL-28B and IL-29 were found at 36–48 h post-infection with CSFV. This finding was further confirmed by independent analysis using quantitative real-time PCR (**Figures 1C,D**), although the increased levels of type III IFNs were limited.

### CSFV Infection Causes Increased Expression of Type III IFNs *in Vivo*

**FIGURE 2:**
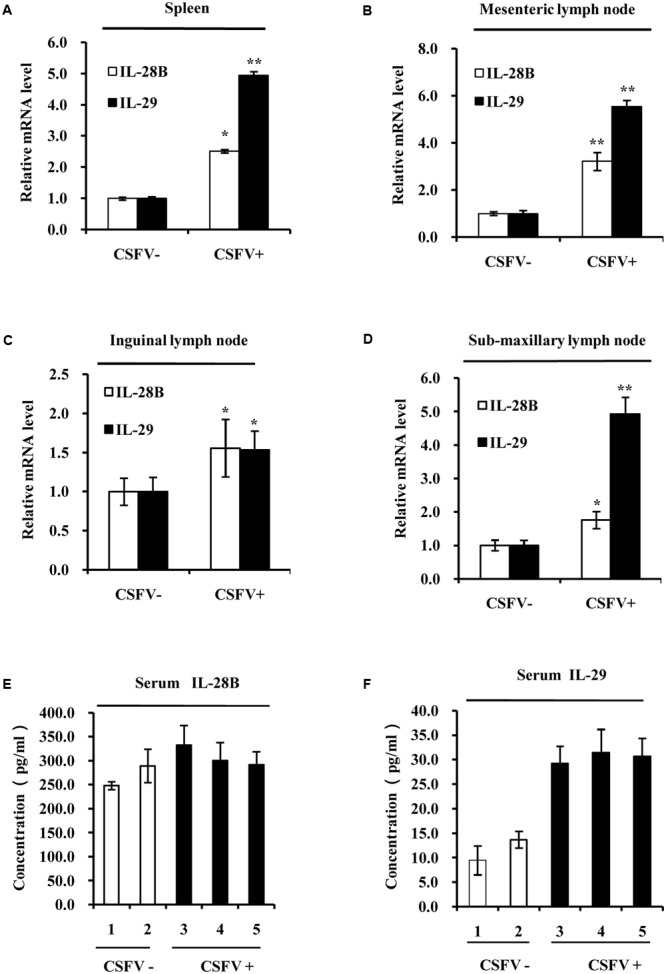
Classical swine fever virus infection increases the expression of type III IFNs in the lymphoid tissues of pigs. Quantitative real-time PCR was performed to examine the mRNA levels of IL-28B and IL-29 in **(A)** spleen, **(B)** mesenteric lymph node, **(C)** inguinal lymph node, and **(D)** sub-maxillary node of pigs infected with CSFV for 5 days and mock treated control pigs. The results are representative of three independent experiments. ^∗^*P* < 0.05, ^∗∗^*P* < 0.01. **(E,F)** Protein levels of IL-28B and IL-29 in the serum of pigs infected with or without CSFV for 5 days were analyzed by ELISA using indicated antibodies. The pigs of numbers 1 and 2 are mock treated controls, and the pigs of numbers 3, 4, and 5 are infected animals by CSFV for 5 days (These are same for other figures). Plotted are the average results from three independent ELISA experiments with similar results.

Next, we determined whether CSFV infection could activate type III IFN response *in vivo*. Pigs were infected with CSFV (0.1 ml per pig, TCID50 = 10^-4^/0.1 ml) and spleen, heart, liver, lungs, kidney, mesenteric lymph node, inguinal lymph node, sub-maxillary lymph node, tonsils, and serum of infected animals were collected 5 days post-infection. We found that high levels of CSFV E2 gene expression were detected in the spleen, mesenteric lymph node, inguinal lymph node, sub-maxillary node, and tonsils after challenge with the virus (Supplementary Figure S2A). Thus, these lymphoid organs were selected for further studies. The expression of swine IL-28B and IL-29 were then examined by RT-PCR (Supplementary Figures S2B–E) and quantitative real-time PCR (**Figures 2A–D**). Using RT-PCR, we observed that expression of IL-28B and IL-29 was clearly induced in spleen, inguinal lymph node, mesenteric lymph node and tonsils of CSFV infected pigs, whereas their expression levels were very low in these organs from mock-treated control animals (Supplementary Figures S2B–E). Similarly, results from analysis using quantitative real-time PCR showed that expression of IL-28B and IL-29 was significantly increased in spleen, mesenteric lymph node, inguinal lymph node, and sub-maxillary node in pigs infected with CSFV as compared to that in these organs of control animals (**Figures 2A–D**). In particular, mRNA levels of IL-29 in spleen, mesenteric lymph node, and sub-maxillary node of the pigs were the most highly elevated after the virus infection (**Figures 2A,B,D**). Furthermore, protein levels of IL-28B and IL-29 were examined in pig serum by ELISA. The data exhibited that IL-29 protein levels were markedly increased in serum of infected pigs as compared with the control animals, but no significant change in IL-28B protein levels was detected in pig serum after infection with the virus (**Figures 2E,F**). Taken together, these experiments demonstrate that infection with virulent CSFV Shimen strain can induce the expression of type III IFNs both *in vitro* and *in vivo*.

### NF-κB Is Involved in Regulating the Expression of Type III IFNs Induced by CSFV

**FIGURE 3:**
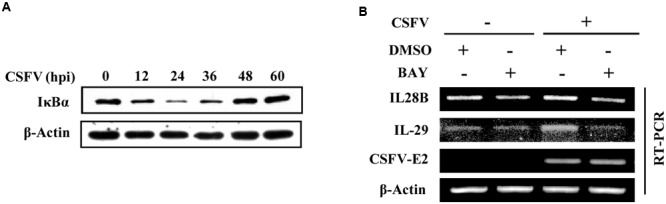
NF-κB is involved in regulation of type III IFNs expression in PK-15 cells infected with CSFV. **(A)** PK-15 cells were infected with CSFV for indicated time, followed by Western blotting with indicated antibodies. **(B)** PK-15 cells were pre-treated with either BAY11- 7082 (NF-κB inhibitor) or DMSO (mock treatment control) for 3 h, and then infected with or without CSFV for 36 h. The cells were harvested and followed by RT-PCR analysis of IL-28B and IL-29 levels. Shown are representative of three independent experiments with similar results.

NF-κB, an inducible transcription factor, plays a variety of evolutionarily conserved roles in the immune system (Hayden and Ghosh, 2014). In an attempt to provide an insight into the mechanism underlying the expression of type III IFNs during CSFV infection, we tested whether there was a functional link between induced expression of type III IFNs and activation of NF-κB, a key transcriptional factor downstream of the interaction between host PRRs and viral PAMPs (Ank et al., 2006b). To this end, cells were infected with CSFV and IκB-α protein was examined. We found that the protein level of IκB-α, an inhibitor of NF-κB, decreased in PK-15 cells at different time points (12–36 h) post-infection (**Figure 3A**). Therefore, we hypothesized that NF-κB might be a key factor in regulating the expression of type III IFNs induced by CSFV. To test this possibility, PK-15 cells were treated with either BAY11-7082 (a selective inhibitor of NF-κB) or DMSO (control) and then infected with CSFV. Indeed, the CSFV-induced expression of IL-28B and IL-29 was reduced in NF-κB inhibited cells (**Figure 3B**). These data suggest that NF-κB may play an important role in regulating the production of IL-28B and IL-29 during the CSFV infection.

### CSFV Infection Activates STAT1 *in Vitro* and *in Vivo*

**FIGURE 4:**
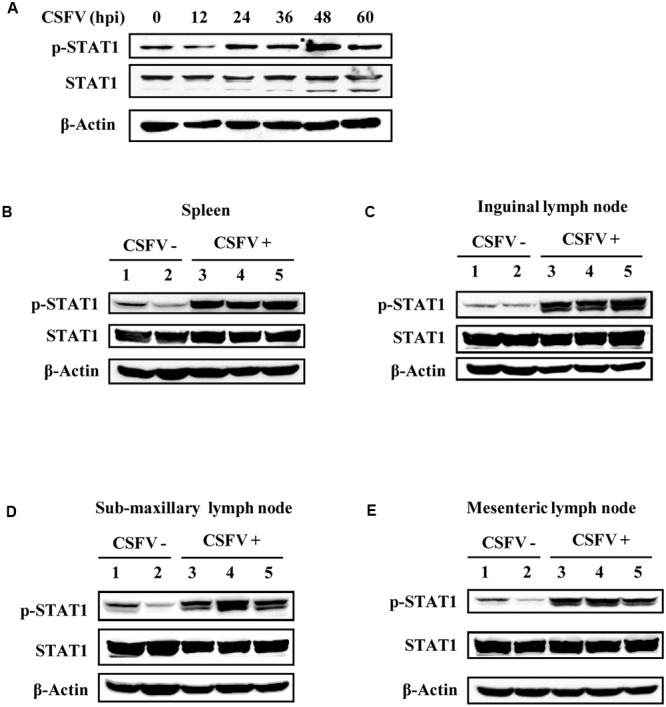
STAT1 is activated by CSFV infection *in vitro* and *in vivo*. **(A)** PK-15 cells were infected with CSFV for indicated time, followed by analysis of Western blotting using indicated antibodies. **(B–E)** Pigs were infected with CSFV for 5 days as described in **Figure 2**, followed by Western blotting using indicated antibodies. Expression of STAT1 and its phosphorylation status were examined in **(B)** spleen, **(C)** inguinal lymph node, **(D)** sub-maxillary node, and **(E)** mesenteric lymph node. Shown are representative of three independent experiments.

In natural host, the strength and the duration of cytokine expression and cytokine-activated signaling are tightly regulated (Cao, 2016). Since type III IFNs were induced during the CSFV infection, we asked whether their downstream signaling was normally activated. Production of type III IFNs is regulated through similar signal transduction pathways as type I IFNs (Ank et al., 2006b; Zhang et al., 2008), and these IFNs primarily activate the JAK-STAT1 signal pathway to achieve their antiviral function (Egli et al., 2014). Therefore, to address this question, we sought to investigate activation of STAT1 during the viral infection. For this, a time course experiment was conducted *in vitro*. We found that phosphorylation of STAT1 in CSFV infected PK-15 cells was up-regulated at early stages of infection (24, 36, and 48 h p.i.), but slightly declined at later stages of infection (after 60 h p.i.) (**Figure 4A**). Furthermore, phosphorylation status of STAT1 during the CSFV infection was examined *in vivo*. Strikingly, phosphorylation levels of STAT1 were greatly elevated in spleen of pigs infected with CSFV as compared to those in control animals (**Figure 4B**). Similar results were also obtained from experiments examining STAT1 phosphorylation in inguinal lymph node of CSFV infected pigs (**Figure 4C**). In addition, we observed that phosphorylation of STAT1 was also increased in sub-maxillary node and mesenteric lymph node in CSFV infected pigs, compared to that in control pigs (**Figures 4D,E**). These data suggest that effective activation of STAT1 signaling can be triggered by the CSFV infection.

### Expression of Some Critical ISGs Is Induced by CSFV Infection *in Vitro* and *in Vivo*

**FIGURE 5:**
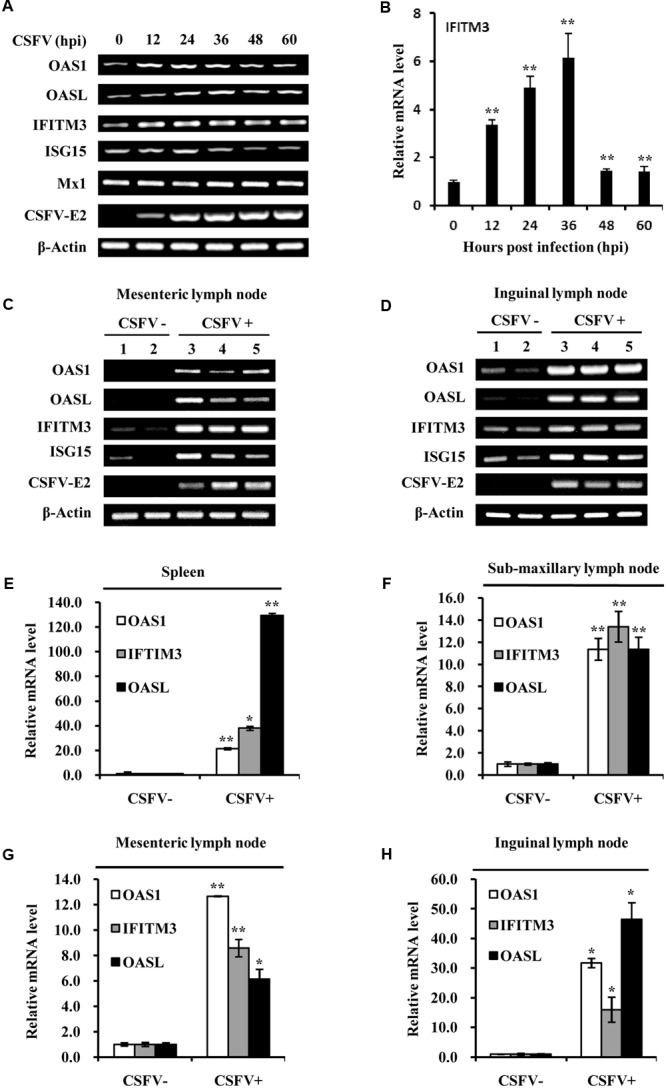
Classical swine fever virus infection elevates the mRNA levels of several critical ISGs *in vitro* and *in vivo*. **(A)** PK-15 cells were infected with or without CSFV and harvested at the indicated time, followed by RT-PCR analysis of indicated genes. **(B)** Quantitative real-time PCR analysis was performed to determine the mRNA levels of IFITM3 in PK-15 cells infected with CSFV for indicated time. Plotted are the average results from three independent experiments. Expression of indicated ISGs and CSFV E2 gene was examined by RT-PCR analysis in **(C)** mesenteric lymph node and **(D)** inguinal lymph node of pigs infected with CSFV for 5 days. Quantitative real-time PCR analysis was performed to examine the mRNA expression of OAS1, OASL, and IFITIM3 in **(E)** spleen, **(F)** sub-maxillary node, **(G)** mesenteric lymph node, and **(H)** inguinal lymph node of pigs infected with CSFV for 5 days. Plotted are the average results from three independent experiments. ^∗^*P* < 0.05, ^∗∗^*P* < 0.01.

As we found that CSFV infection induced the expression of type III IFNs and activation of STAT1, next we further tested whether CSFV infection could enhance the expression of some key ISGs *in vitro* and *in vivo*. For this, RT-PCR and quantitative real-time PCR was performed. As shown in **Figure 5A**, the mRNA expression of some key ISGs, including IFITM3, OAS1 and OASL, was induced in PK-15 cells during the CSFV infection. Expression of ISG15 was slightly increased at early stages during the CSFV infection, but the virus infection had no significant effect on expression of Mx1 (**Figure 5A**). The increased mRNA expression of IFITM3, OAS1, OASL, and ISG15 in infected PK-15 cells was further confirmed by quantitative real-time PCR (**Figure 5B** and Supplementary Figure S3). mRNA expression of these ISGs was then examined *in vivo*. Using RT-PCR, we observed that expression of IFITM3, ISG15, OAS1, and OASL was markedly up-regulated in spleen, mesenteric lymph node, inguinal lymph node, sub-maxillary node, and tonsils in pigs infected with CSFV as compared to that in the control animals (**Figures 5C,D** and Supplementary Figure S4). Similarly, analysis by quantitative real-time PCR showed that expression of IFITM3, OAS1, and OASL was significantly increased in spleen, mesenteric lymph node, inguinal lymph node, and sub-maxillary node of infected pigs as compared with the controls (**Figures 5E–H**).

**FIGURE 6:**
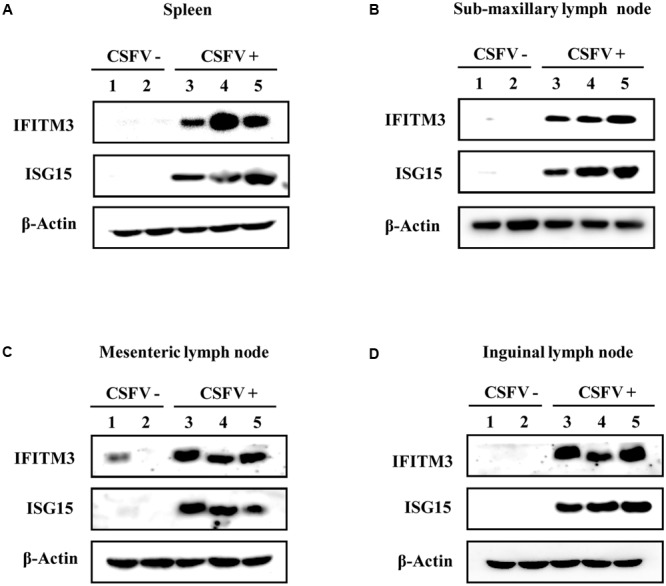
Protein expression of IFITM3 and ISG15 in pigs is up-regulated by CSFV infection. **(A–D)** Pigs were infected with CSFV (numbers 3, 4, and 5) or mock treatment (numbers 1 and 2) for 5 days, followed by Western blotting using indicated antibodies. Shown is the expression of IFITM3 and ISG15 in **(A)** spleen, **(B)** sub-maxillary node, **(C)** mesenteric lymph node, and **(D)** inguinal lymph node. Shown are representative of three independent experiments with similar results.

Moreover, protein expression of IFITM3 and ISG15 in pigs infected with CSFV was examined by Western blot analysis (**Figures 6A–D**). Indeed, we observed that IFITM3 and ISG15 protein levels were greatly elevated in spleen, mesenteric lymph node, inguinal lymph node, and sub-maxillary node in pigs after the infection (**Figures 6A–D**). Together, these results suggest that CSFV infection can trigger the innate immune response in pigs, which may play an important role in host defense against the virus infection in the early days.

## Discussion

Classical swine fever causes huge economic losses in pig industry particularly in developing countries (Li et al., 2013). Understanding pathogenesis and host antiviral response is critical for finding a feasible control strategy against CSFV infection. However, so far, the pathogenesis of CSFV and the host antiviral immune response are still not fully determined. In the present study, we investigated the involvement of host innate immunity by assessing the expression of type III IFNs and activation of their downstream signaling, as well as expression of several critical ISGs after infection of PK-15 cells and pigs with CSFV Shimen strain. We observed that CSFV infection induced the expression of type III IFNs, enhanced the phosphorylation of STAT1, and caused up-regulation of several critical ISGs in PK-15 cells and pigs after the infection. The kinetics of type III IFNs, the ISGs and STAT1 activation in lymphoid tissues of pigs after the exposure to CSFV were similar to those in PK-15 cells, but their quantity appeared to be higher in the infected animals. These data indicate that CSFV can effectively activate the host innate immune system and thereby initiates a positive antiviral immune response both *in vitro* and *in vivo*.

It has been shown that infection with several single-stranded RNA viruses can induce the expression of type III IFNs (Brand et al., 2005). However, Type III IFNs can only be produced by specific cell types such as lymphoid cells, in contrast to type I IFNs which can be expressed by almost all cell types (Ank et al., 2006a). The interferon signaling pathways and their regulation are very complex and diverse. Here, we found that CSFV infection induced the expression of Type III IFNs, but the increased levels of type III IFNs caused by the virus were limited as compared to their expression levels induced by influenza A virus in our previous studies (Wei et al., 2014). These observations suggest that CSFV may have evolved a strategy to suppress the expression of type III IFNs. It is known that type III IFNs are induced through similar signal transduction pathways as type I IFNs (Ank et al., 2006b; Zhang et al., 2008), and both type I and type III IFNs activate the JAK-STAT signal pathway to achieve their antiviral function (Egli et al., 2014). For example, activation of signaling pathways mediated by TLR-3 and RIG-I, which have been well-characterized to induce IFN-α/β production, also induces type III IFNs in murine and human cells (Dumoutier et al., 2004; Onoguchi et al., 2007; Zhou et al., 2009). But unlike type I IFN receptor, type III IFN receptor is expressed in a cell-specific fashion (Sommereyns et al., 2008). It still remains to be determined how expression of type III IFNs is regulated and how they activate STAT1 in a cell-specific fashion during the CSFV infection.

Many viruses have potential to trigger the production of interferons to a certain extent. Previous studies have shown that a number of RNA viruses are able to trigger the expression of Type III IFNs (Coccia et al., 2004; Ank et al., 2006a). To establish a successful infection, however, RNA viruses can interrupt signaling pathways and perturb host antiviral defenses to evade the innate immunity. Limited induction of type III IFNs indicates that there exists a mechanism by which CSFV inhibits the signaling pathway regulating expression of type III IFNs. Therefore, it is of interest to study the mechanism underlying control of type III IFNs by CSFV. On the other hand, to combat the infection, host immune system deploys various antiviral cytokines that can activate a number of key downstream signaling pathways like JAK/STAT, which increases the production of effective antiviral molecules, including numerous ISGs (Takeuchi and Akira, 2009). In this study, we found that STAT1 was clearly activated in PK-15 cells following the CSFV infection. Furthermore, we observed that STAT1 was greatly activated in pigs infected with CSFV. It was previously revealed that CSFV infection caused an increased production of type I IFNs (Dong et al., 2013). Together, these results suggest that both type I and type III IFNs likely contribute to activation of STAT1 during the CSFV infection.

Further analysis determined that NF-κB is involved in regulation of the CSFV-induced expression of type III IFNs. It is well-known that after recognizing the viral RNA, RIG-I and MDA-5 interact with CARD and IPS-1 to recruit TRAF6, TBK1 and IKK, which activate the downstream signaling of transcription factors IRFs and NF-κB (Han et al., 2014; Goraya et al., 2015). A previous study revealed that CSFV was recognized by RIG-I and MDA-5 and activated the NF-κB signaling pathway, ultimately resulting in production of type I IFNs (Dong et al., 2013). NF-κB acts as a transcription factor that responds to cellular environment upon viral infection and controls the expression of innate molecules (Bonizzi and Karin, 2004). IκBα is a specific inhibitor of NF-κB, which interacts with NF-κB dimers and retains NF-κB in the cytoplasm of different cell types (Karin and Ben-Neriah, 2000). Degradation of the IκBα subunit leads to the release and translocation of NF-κB to the nucleus, which activates the transcription of IFN genes involved in innate immunity (Kanarek et al., 2010). In the present study, we found that the levels of IκBα decreased in response to CSFV infection, suggesting an increase of NF-κB activity. In order to elucidate the function of NF-κB in response to CSFV infection, cells were treated with BAY11, a NF-κB inhibitor, prior to CSFV infection. Interestingly, our experiments demonstrated that expression of IL-28B and IL-29 was significantly reduced in CSFV-infected cells after treatment with BAY11. Together, these findings suggest that NF-κB may play an important role in antiviral innate immunity against CSFV infection.

Type III IFNs functionally resemble type I IFNs, inducing antiviral protection *in vitro* (Kotenko et al., 2003; Robek et al., 2005) as well as *in vivo* (Ank et al., 2006b). Interaction between type III IFNs and their receptor enhances the phosphorylation of STAT family and the formation of the interferon-stimulated gene factor 3 (ISGF3) complex (Kotenko et al., 2003), and the induction of typical IFN-induced genes like the IFITM3, OAS1, OASL, and Mx1 genes (Sadler and Williams, 2008; Li et al., 2016). In the present study, we noticed that expression of several critical ISGs including IFITM3, OASL, OAS1, and ISG15 was upregulated in both PK-15 cells and lymphoid tissues of pigs infected with CSFV. A previous study revealed that OASL suppressed the CSFV replication via interacting with MDA-5, a double stranded RNA sensor, and enhanced type I IFN signaling (Li et al., 2017). However, precise roles of other ISGs in the innate immunity against CSFV are needed to be elucidated.

We found that expression levels of OAS1, IFTIM3, and OASL in CSFV infected pigs and control animals showed some variation among spleen, mesenteric lymph node, sub-maxillary lymph node and inguinal lymph node. It is likely that this is due to varied cytotropism of CSFV in pigs. Although we observed basal expression of IFITM3 and ISG15 in mesenteric lymph node and inguinal lymph node of mock infected animals, significant increase in the expression of these ISGs was seen in these lymphoid tissues of CSFV infected pigs as compared to that in the control animals. Interestingly, there was an unexpected expression of IFITM3 at a low level observed in a negative control pig. These data suggest that there is a possibility that expression levels of particular ISGs might be varied due to individual differences. Therefore, a large number of the animals might be needed in further studies.

In addition, suppressor of cytokine signaling (SOCS) proteins play a critical role in the regulation of JAK/STAT signaling pathway (Shuai and Liu, 2003). They specifically bind with key phosphorylated tyrosine residues in the activation site on JAK proteins. SOCS proteins also bind with cytokine receptors and degrade signaling molecules to negatively regulate JAK/STAT signaling (Strebovsky et al., 2012). Therefore, more studies are required to reveal the biological significance of SOCS proteins in regulation of JAK/STAT pathway and expression of ISGs in response to CSFV infection.

## Author Contributions

BxC, QB, and XC performed the most experiments and wrote the manuscript. J-LC, MG, LW, and BC performed some experiments and analyzed the data. SW and XC analyzed the data and revised the manuscript. J-LC designed the study, wrote and revised the manuscript. All authors read and approved the final manuscript.

## Conflict of Interest Statement

The authors declare that the research was conducted in the absence of any commercial or financial relationships that could be construed as a potential conflict of interest. The reviewer GS and handling Editor declared their shared affiliation.

## References

[B1] Ambagala A. P. Solheim J. C. Srikumaran S. (2005). Viral interference with MHC class I antigen presentation pathway: the battle continues. *Vet. Immunol. Immunopathol.* 107 1–15. 10.1016/j.vetimm.2005.04.006 15978672

[B2] Ank N. West H. Bartholdy C. Eriksson K. Thomsen A. R. Paludan S. R. (2006a). Lambda interferon (IFN-lambda), a type III IFN, is induced by viruses and IFNs and displays potent antiviral activity against select virus infections in vivo. *J. Virol.* 80 4501–4509. 16611910 10.1128/JVI.80.9.4501-4509.2006 PMC1472004

[B3] Ank N. West H. Paludan S. R. (2006b). IFN-lambda: novel antiviral cytokines. *J. Interferon Cytokine Res.* 26 373–379. 10.1089/jir.2006.26.373 16734557

[B4] Bonizzi G. Karin M. (2004). The two NF-kappaB activation pathways and their role in innate and adaptive immunity. *Trends Immunol.* 25 280–288. 10.1016/j.it.2004.03.008 15145317

[B5] Borden E. C. Sen G. C. Uze G. Silverman R. H. Ransohoff R. M. Foster G. R. (2007). Interferons at age 50: past, current and future impact on biomedicine. *Nat. Rev. Drug Discov.* 6 975–990. 10.1038/nrd2422 18049472 PMC7097588

[B6] Brand S. Beigel F. Olszak T. Zitzmann K. Eichhorst S. T. Otte J.-M. (2005). IL-28A and IL-29 mediate antiproliferative and antiviral signals in intestinal epithelial cells and murine CMV infection increases colonic IL-28A expression. *Am. J. Physiol. Gastrointest. Liver Physiol.* 289 G960–G968. 10.1152/ajpgi.00126.2005 16051921

[B7] Cao X. (2016). Self-regulation and cross-regulation of pattern-recognition receptor signalling in health and disease. *Nat. Rev. Immunol.* 16 35–50. 10.1038/nri.2015.8 26711677

[B8] Cao Z. Guo K. Zheng M. Ning P. Li H. Kang K. (2015). A comparison of the impact of Shimen and C strains of classical swine fever virus on Toll-like receptor expression. *J. Gen. Virol.* 96(Pt. 7) 1732–1745. 10.1099/vir.0.000129 25805409

[B9] Chen Z. Luo G. Wang Q. Wang S. Chi X. Huang Y. (2015). Muscovy duck reovirus infection rapidly activates host innate immune signaling and induces an effective antiviral immune response involving critical interferons. *Vet. Microbiol.* 175 232–243. 10.1016/j.vetmic.2014.12.004 25554243

[B10] Coccia E. M. Severa M. Giacomini E. Monneron D. Remoli M. E. Julkunen I. (2004). Viral infection and Toll-like receptor agonists induce a differential expression of type I and λ interferons in human plasmacytoid and monocyte-derived dendritic cells. *Eur. J. Immunol.* 34 796–805. 10.1002/eji.200324610 14991609

[B11] Diebold S. S. Kaisho T. Hemmi H. Akira S. Reis e Sousa C. (2004). Innate antiviral responses by means of TLR7-mediated recognition of single-stranded RNA. *Science* 303 1529–1531. 10.1126/science.1093616 14976261

[B12] Dong X. Y. Liu W. J. Zhao M. Q. Wang J. Y. Pei J. J. Luo Y. W. (2013). Classical swine fever virus triggers RIG-I and MDA5-dependent signaling pathway to IRF-3 and NF-kappaB activation to promote secretion of interferon and inflammatory cytokines in porcine alveolar macrophages. *Virol. J.* 10:286. 10.1186/1743-422X-10-286 24034559 PMC3849481

[B13] Dumoutier L. Tounsi A. Michiels T. Sommereyns C. Kotenko S. V. Renauld J. C. (2004). Role of the interleukin (IL)-28 receptor tyrosine residues for antiviral and antiproliferative activity of IL-29/interferon-lambda 1: similarities with type I interferon signaling. *J. Biol. Chem.* 279 32269–32274. 10.1074/jbc.M404789200 15166220

[B14] Egli A. Santer D. M. O’Shea D. Tyrrell D. L. Houghton M. (2014). The impact of the interferon-lambda family on the innate and adaptive immune response to viral infections. *Emerg. Microbes Infect.* 3:e51. 10.1038/emi.2014.51 26038748 PMC4126180

[B15] Goraya M. U. Wang S. Munir M. Chen J.-L. (2015). Induction of innate immunity and its perturbation by influenza viruses. *Protein Cell* 6 712–721. 10.1007/s13238-015-0191-z 26206138 PMC4598321

[B16] Han Y. W. Choi J. Y. Uyangaa E. Kim S. B. Kim J. H. Kim B. S. (2014). Distinct dictation of Japanese encephalitis virus-induced neuroinflammation and lethality via triggering TLR3 and TLR4 signal pathways. *PLOS Pathog.* 10:e1004319. 10.1371/journal.ppat.1004319 25188232 PMC4154777

[B17] Hayden M. S. Ghosh S. (2014). Regulation of NF-kappaB by TNF family cytokines. *Semin. Immunol.* 26 253–266. 10.1016/j.smim.2014.05.004 24958609 PMC4156877

[B18] Iwasaki A. Medzhitov R. (2010). Regulation of adaptive immunity by the innate immune system. *Science (New York, N.Y.)* 327 291–295. 10.1126/science.1183021 20075244 PMC3645875

[B19] Janeway C. A. Jr. Medzhitov R. (2002). Innate immune recognition. *Annu. Rev. Immunol.* 20 197–216. 10.1146/annurev.immunol.20.083001.084359 11861602

[B20] Ji W. Guo Z. Ding N. Z. He C. Q. (2015). Studying classical swine fever virus: making the best of a bad virus. *Virus Res.* 197 35–47. 10.1016/j.virusres.2014.12.006 25510481

[B21] Kanarek N. London N. Schueler-Furman O. Ben-Neriah Y. (2010). Ubiquitination and degradation of the inhibitors of NF-kappaB. *Cold Spring Harb. Perspect. Biol.* 2:a000166. 10.1101/cshperspect.a000166 20182612 PMC2828279

[B22] Karin M. Ben-Neriah Y. (2000). Phosphorylation meets ubiquitination: the control of NF-[kappa]B activity. *Annu. Rev. Immunol.* 18 621–663. 10.1146/annurev.immunol.18.1.621 10837071

[B23] Kawai T. Akira S. (2006). TLR signaling. *Cell Death Diff.* 13 816–825. 10.1038/sj.cdd.4401850 16410796

[B24] Knoetig S. M. Summerfield A. Spagnuolo-Weaver M. McCullough K. C. (1999). Immunopathogenesis of classical swine fever: role of monocytic cells. *Immunology* 97 359–366. 10.1046/j.1365-2567.1999.00775.x 10447754 PMC2326829

[B25] Kotenko S. V. Gallagher G. Baurin V. V. Lewis-Antes A. Shen M. Shah N. K. (2003). IFN-lambdas mediate antiviral protection through a distinct class II cytokine receptor complex. *Nat. Immunol.* 4 69–77. 10.1038/ni875 12483210

[B26] Leifer I. Ruggli N. Blome S. (2013). Approaches to define the viral genetic basis of classical swine fever virus virulence. *Virology* 438 51–55. 10.1016/j.virol.2013.01.013 23415391

[B27] Li D. Li S. Sun Y. Dong H. Li Y. Zhao B. (2013). Poly(C)-binding protein 1, a novel N(pro)-interacting protein involved in classical swine fever virus growth. *J. Virol.* 87 2072–2080. 10.1128/JVI.02807-12 23221550 PMC3571455

[B28] Li F. Chen Y. Zhang Z. Ouyang J. Wang Y. Yan R. (2015). Robust expression of vault RNAs induced by influenza A virus plays a critical role in suppression of PKR-mediated innate immunity. *Nucleic Acids Res.* 43 10321–10337. 10.1093/nar/gkv1078 26490959 PMC4666359

[B29] Li L. F. Yu J. Li Y. Wang J. Li S. Zhang L. (2016). Guanylate-binding protein 1, an interferon-induced GTPase, exerts an antiviral activity against classical swine fever virus depending on its GTPase activity. *J. Virol.* 90 4412–4426. 10.1128/JVI.02718-15 26889038 PMC4836331

[B30] Li L. F. Yu J. Zhang Y. Yang Q. Li Y. Zhang L. (2017). Interferon-inducible oligoadenylate synthetase-like protein acts as an antiviral effector against classical swine fever virus via the MDA5-mediated type I interferon-signaling pathway. *J. Virol.* 91 e01514-16. 10.1128/JVI.01514-16 28331099 PMC5432864

[B31] Lund J. M. Alexopoulou L. Sato A. Karow M. Adams N. C. Gale N. W. (2004). Recognition of single-stranded RNA viruses by Toll-like receptor 7. *Proc. Natl. Acad. Sci. U.S.A.* 101 5598–5603. 10.1073/pnas.0400937101 15034168 PMC397437

[B32] Meyers G. Rumenapf T. Thiel H. J. (1989). Molecular cloning and nucleotide sequence of the genome of hog cholera virus. *Virology* 171 555–567. 10.1016/0042-6822(89)90625-9 2763466

[B33] Meyers G. Thiel H. J. (1996). Molecular characterization of pestiviruses. *Adv. Virus Res.* 47 53–118. 10.1016/S0065-3527(08)60734-4 8895831

[B34] Moennig V. Floegel-Niesmann G. Greiser-Wilke I. (2003). Clinical signs and epidemiology of classical swine fever: a review of new knowledge. *Vet. J.* 165 11–20. 10.1016/S1090-0233(02)00112-0 12618065

[B35] Onoguchi K. Yoneyama M. Takemura A. Akira S. Taniguchi T. Namiki H. (2007). Viral infections activate types I and III interferon genes through a common mechanism. *J. Biol. Chem.* 282 7576–7581. 10.1074/jbc.M608618200 17204473

[B36] Ouyang J. Zhu X. Chen Y. Wei H. Chen Q. Chi X. (2014). NRAV, a long noncoding RNA, modulates antiviral responses through suppression of interferon-stimulated gene transcription. *Cell Host Microbe* 16 616–626. 10.1016/j.chom.2014.10.001 25525793 PMC7104942

[B37] Qu L. McMullan L. K. Rice C. M. (2001). Isolation and characterization of noncytopathic pestivirus mutants reveals a role for nonstructural protein NS4B in viral cytopathogenicity. *J. Virol.* 75 10651–10662. 10.1128/jvi.75.22.10651-10662.2001 11602707 PMC114647

[B38] Robek M. D. Boyd B. S. Chisari F. V. (2005). Lambda interferon inhibits hepatitis B and C virus replication. *J. Virol.* 79 3851–3854. 10.1128/JVI.79.6.3851-3854.2005 15731279 PMC1075734

[B39] Rossi S. Toigo C. Hars J. Pol F. Hamann J.-L. Depner K. (2011). New insights on the management of wildlife diseases using multi-state recapture models: the case of classical swine fever in wild boar. *PLOS ONE* 6:e24257. 10.1371/journal.pone.0024257 21977225 PMC3178526

[B40] Sadler A. J. Williams B. R. (2008). Interferon-inducible antiviral effectors. *Nat. Rev. Immunol.* 8 559–568. 10.1038/nri2314 18575461 PMC2522268

[B41] Sen G. C. Sarkar S. N. (2005). Transcriptional signaling by double-stranded RNA: role of TLR3. *Cytokine Growth Factor Rev.* 16 1–14. 10.1016/j.cytogfr.2005.01.006 15733829

[B42] Shuai K. Liu B. (2003). Regulation of JAK-STAT signalling in the immune system. *Nat. Rev. Immunol.* 3 900–911. 10.1038/nri1226 14668806

[B43] Sommereyns C. Paul S. Staeheli P. Michiels T. (2008). IFN-lambda (IFN-lambda) is expressed in a tissue-dependent fashion and primarily acts on epithelial cells in vivo. *PLOS Pathog.* 4:e1000017. 10.1371/journal.ppat.1000017 18369468 PMC2265414

[B44] Strebovsky J. Walker P. Dalpke A. H. (2012). Suppressor of cytokine signaling proteins as regulators of innate immune signaling. *Front. Biosci.* 17 1627–1639. 10.2741/4008 22201825

[B45] Summerfield A. Hofmann M. A. McCullough K. C. (1998). Low density blood granulocytic cells induced during classical swine fever are targets for virus infection. *Vet. Immunol. Immunopathol.* 63 289–301. 10.1016/S0165-2427(98)00108-1 9656461

[B46] Susa M. Konig M. Saalmuller A. Reddehase M. J. Thiel H. J. (1992). Pathogenesis of classical swine fever: B-lymphocyte deficiency caused by hog cholera virus. *J. Virol.* 66 1171–1175. 1731095 10.1128/jvi.66.2.1171-1175.1992 PMC240821

[B47] Takaoka A. Yanai H. (2006). Interferon signalling network in innate defence. *Cell Microbiol.* 8 907–922. 10.1111/j.1462-5822.2006.00716.x 16681834

[B48] Takeuchi O. Akira S. (2009). Innate immunity to virus infection. *Immunol. Rev.* 227 75–86. 10.1111/j.1600-065X.2008.00737.x 19120477 PMC5489343

[B49] Terpstra C. (1987). Epizootiology of swine fever. *Vet. Q.* 9(Suppl. 1) 50S–60S. 10.1080/01652176.1987.9694138 3324456

[B50] Wang S. Chi X. Wei H. Chen Y. Chen Z. Huang S. (2014). Influenza A virus-induced degradation of eukaryotic translation initiation factor 4B contributes to viral replication by suppressing IFITM3 protein expression. *J. Virol.* 88 8375–8385. 10.1128/JVI.00126-14 24829357 PMC4135930

[B51] Wei H. Wang S. Chen Q. Chen Y. Chi X. Zhang L. (2014). Suppression of interferon lambda signaling by SOCS-1 results in their excessive production during influenza virus infection. *PLOS Pathog.* 10:e1003845. 10.1371/journal.ppat.1003845 24391501 PMC3879354

[B52] Yoneyama M. Kikuchi M. Matsumoto K. Imaizumi T. Miyagishi M. Taira K. (2005). Shared and unique functions of the DExD/H-box helicases RIG-I, MDA5, and LGP2 in antiviral innate immunity. *J. Immunol.* 175 2851–2858. 10.4049/jimmunol.175.5.2851 16116171

[B53] Zhang S. Y. Boisson-Dupuis S. Chapgier A. Yang K. Bustamante J. Puel A. (2008). Inborn errors of interferon (IFN)-mediated immunity in humans: insights into the respective roles of IFN-alpha/beta, IFN-gamma, and IFN-lambda in host defense. *Immunol. Rev.* 226 29–40. 10.1111/j.1600-065X.2008.00698.x 19161414

[B54] Zhou L. Wang X. Wang Y. J. Zhou Y. Hu S. Ye L. (2009). Activation of Toll-like receptor-3 induces interferon-λ expression in human neuronal cells. *Neuroscience* 159 629–637. 10.1016/j.neuroscience.2008.12.036 19166911 PMC2650740

