# Supplementary material for: Infection with Classical Swine Fever Virus Induces Expression of Type III Interferons and Activates Innate Immune Signaling
Source: Front Microbiol. 2017 Dec 19;8:2558. doi: 10.3389/fmicb.2017.02558 (PMC5742159; doi:10.3389/fmicb.2017.02558)
Supplement: Supplementary file 1 [file Data_Sheet_1.PDF]

Supplementary Figure Legends

Figure S1. CSFV is well replicated in PK-15 cells as indicated by robust expression of the viral E2 gene.

Quantitative real-time PCR analysis was performed to examine the mRNA levels of CSFV E2, which encodes one of CSFV structural protein, in PK-15 cells infected with CSFV for indicated times. Plotted are the average results from three independent experiments.

Figure S2. CSFV infection induces the expression of type III IFNs in the lymphoid tissues of pigs.

(A) Pigs were infected with CSFV for 5 days, and then various lymphoid tissues (spleen, tonsils, inguinal lymph node, sub-maxillary lymph node, and mesenteric lymph node) were collected, followed by RT-PCR to examine the CSFV E2 mRNA expression. (B-E) RT-PCR was performed to examine the IL-28B and IL-29 mRNA expression in (B) spleen, (C) inguinal lymph node, (D) mesenteric lymph node, and (E) tonsils of pigs infected with CSFV or mock treatment for 5 days.

Figure S3. CSFV infection upregulates the expression of several critical ISGs in PK-15 cells.

(A-C) Quantitative real-time PCR analysis was performed to examine the mRNA levels of (A) OAS1, (B) OASL, and (C) ISG15 in PK-15 cells infected with CSFV for indicated times. Plotted are the average results from three independent experiments.

Figure S4. Expression of several ISGs is upregulated by CSFV infection *in vivo*.

RT-PCR analysis was performed to determine the mRNA expression of indicated ISGs in (A) spleen, (B) tonsils, and (C) sub-maxillary lymph node of pigs infected with CSFV or mock treatment for 5 days.
