# Supplementary figures and images for: Infection with Classical Swine Fever Virus Induces Expression of Type III Interferons and Activates Innate Immune Signaling
Source: Front Microbiol. 2017 Dec 19;8:2558. doi: 10.3389/fmicb.2017.02558 (PMC5742159; doi:10.3389/fmicb.2017.02558)

Figure S1

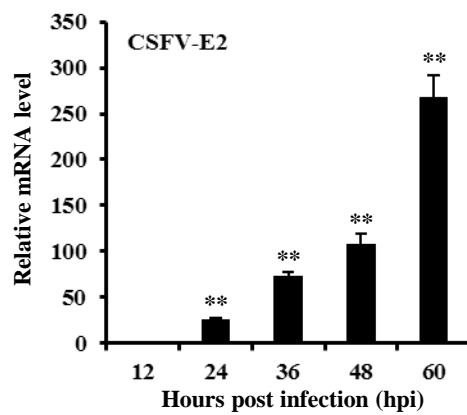

Figure S2

A

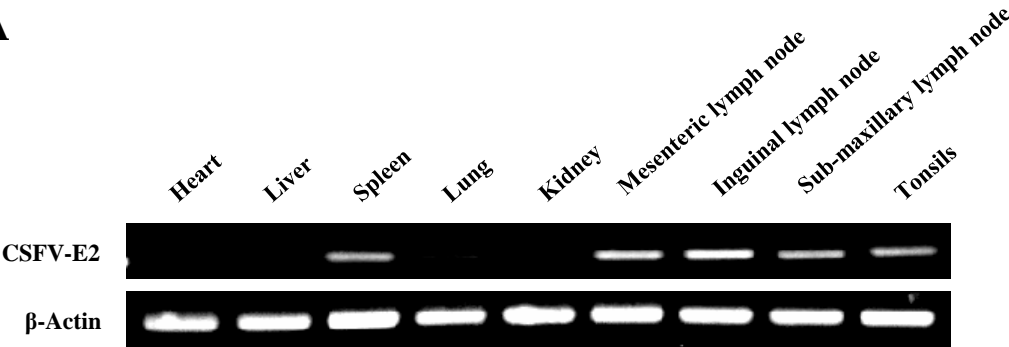

B

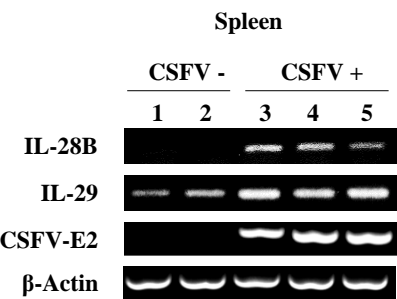

C

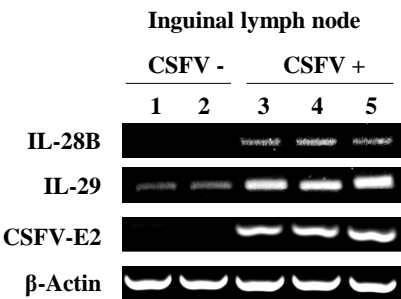

D

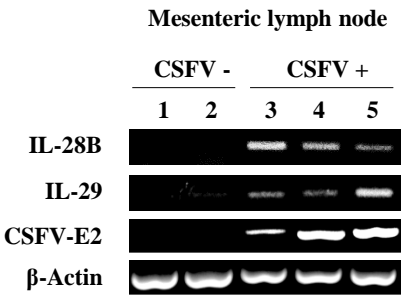

E

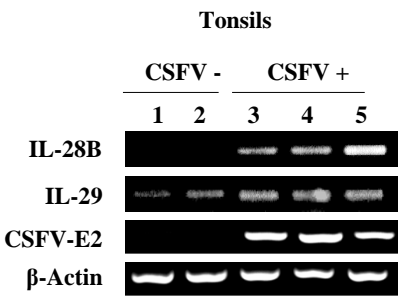

Figure S3

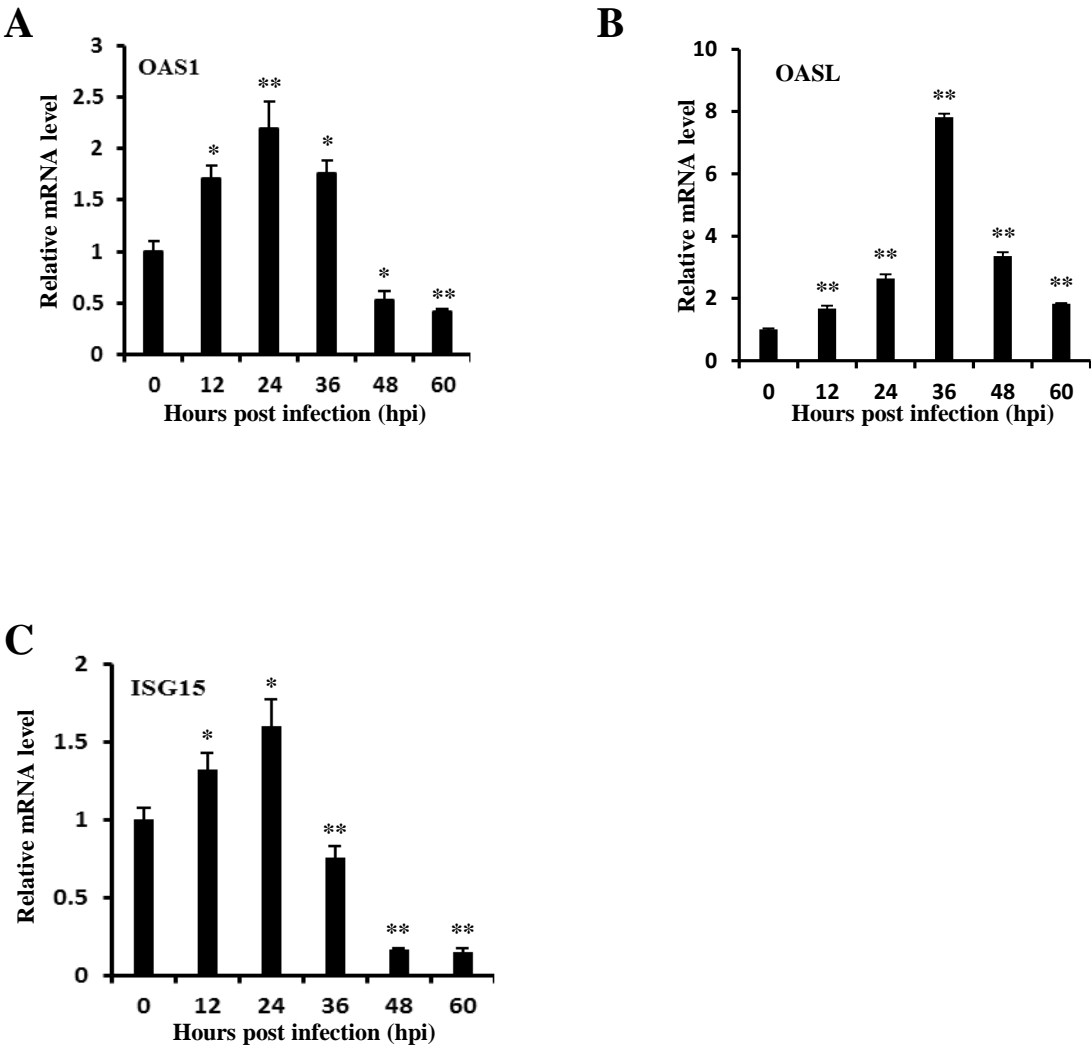

Figure S4

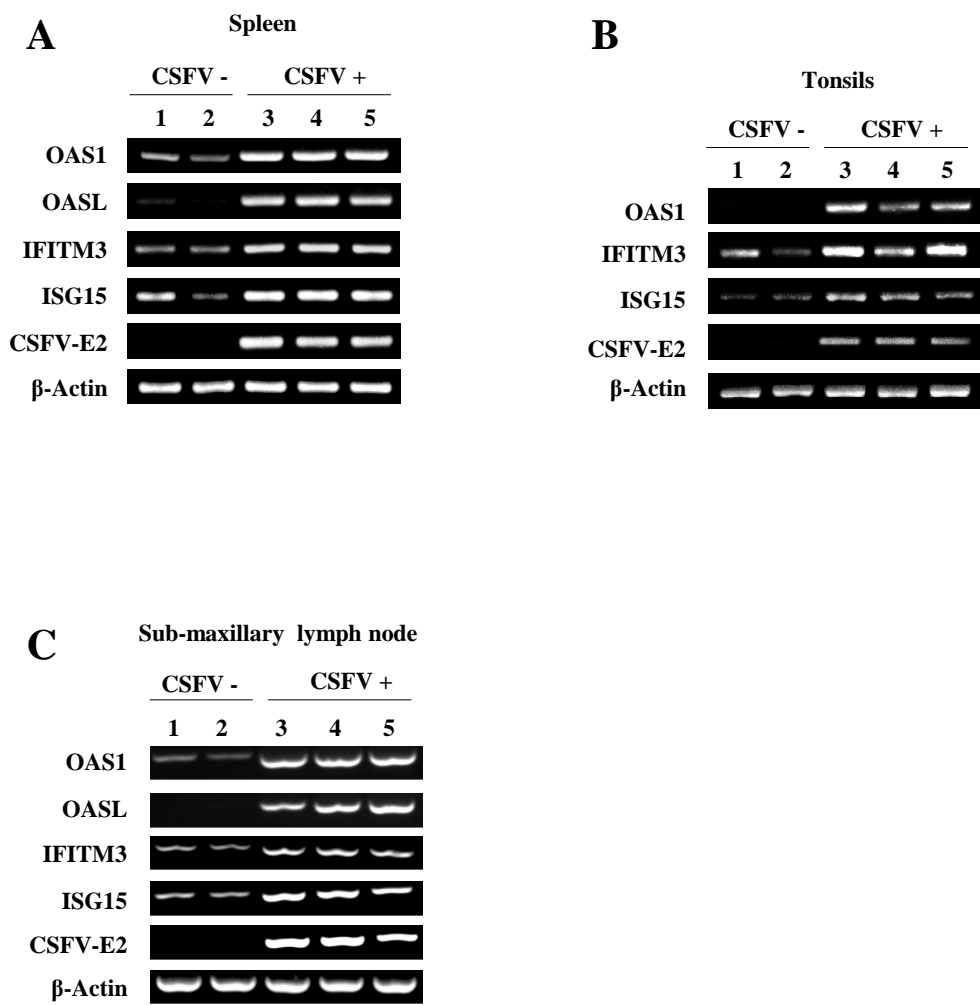

Supplement: Supplementary file 2 [file Data_Sheet_2.PDF]
